# Palladium Phthalocyanine Nanowire-Based Highly Sensitive Sensors for NO_2(g)_ Detection

**DOI:** 10.3390/s24061819

**Published:** 2024-03-12

**Authors:** Crystal Otero Vélez, Soraya Y. Flores, Luis F. Fonseca, Dalice M. Piñero Cruz

**Affiliations:** 1 Faculty of Natural Sciences, University of Puerto Rico, Río Piedras Campus, San Juan 00931, Puerto Rico; crystal.otero1@upr.edu (C.O.V.); soraya.flores@upr.edu (S.Y.F.); luis.fonseca@upr.edu (L.F.F.); 2 Molecular Science Research Center, San Juan 00926-2614, Puerto Rico

**Keywords:** palladium phthalocyanine, nanowires, chemiresistors, gas sensors, nitrogen dioxide

## Abstract

Palladium phthalocyanine (PdPc) nanowires (NWs) were developed to achieve the gas sensing of NO_2_ in the sub-parts-per-million (ppm) range. Non-substituted metal phthalocyanine are well known for their p-type semiconducting behavior, which is responsible for its gas-sensing capabilities. Nanofabrication of the PdPc NWs was performed by physical vapor deposition (PVD) on an interdigitated gold electrode (IDE). The coordination of palladium in the structure was confirmed with UV–Vis spectroscopy. Gas-sensing experiments for NO_2_ detection were undertaken at different sensed gas concentrations from 4 ppm to 0.5 ppm at room temperature. In this work, the responses at different gas concentrations are reported. In addition, structural studies of the PdPc NWs with scanning electron microscopy (SEM) and electron-dispersive X-ray diffraction (EDS) are shown.

## 1. Introduction

Nitrogen dioxide (NO_2_) is a pollutant gas found mainly where combustion processes occur. NO_2_ is categorized as a toxic gas dangerous to human health when being exposed to concentrations of 0.12 ppm for periods longer than one hour [1]. Detecting NO_2_ in the environment is of extreme importance considering the detrimental effects on the respiratory system. Prolonged exposure can result in serious respiratory diseases, such as bronchitis and asthma [1,2]. Anthropogenic sources of NO_2_ include combustion processes that emit nitrogen monoxide (NO). Following the emission, NO reacts with the oxygen molecules present in the environment, where it then oxidizes to form NO_2_ [3]. Common NO producers are found in urban locations where high volumes of combustion vehicles are the most abundant source of air pollution. Environmental risks of high concentrations of NO_2_ include reduced visibility, possibility of acid rain and affecting the vegetation growth [4]. Other NO_2_ sources include space exploration launching applications. Rocket propulsion systems can emit NO_2_ if there is a gas leak in the liquid rocket arrangement. The components of these propulsion systems include the propellants, fuel (molecular hydrogen) and an oxidizer (N_2_O_4_). A leakage of nitrogen tetroxide (N_2_O_4_) produces NO_2_ that can be detected using a chemical sensor [5]. Gas sensors with a fast response are crucial for preventing toxic gas exposure. When developing a cost-effective and highly functional sensor, there are two key factors to consider. The first factor is the synthesis process of the material. Minimizing the number of steps and the utilization of additional starting materials to reduce monetary requirements is essential. The second factor to consider is the practical application of the sensor. Many commonly used gas detection sensors require additional conditioning, such as a specific temperature or UV exposure to facilitate sensor recovery [6].

An excellent material that can be used to create sensors at working room temperature with a single-step synthesis and practical device preparation are metal phthalocyanines (MPcs). They possess a highly stable structure that when combined with their semiconducting properties provides a favorable choice for gas-sensing device creation. In the last decade, MPcs have been used as key materials to develop novel sensitive chemiresistors for a variety of analytes. They are particularly attractive for gas sensing because their sensitivity and response toward various analytes can be tailored by (i) the nature of the central metal atom and (ii) the substituents in the aromatic rings [7,8]. MPcs are macrocyclic organic compounds with a conjugated planar structure composed of 18 π-electrons that are responsible for interactions with incoming gases that result in sensing responses [7,8]. Peripheral and non-peripheral positions on the external rings allow for the incorporation of different substituents that alter the material’s semiconducting properties. MPc semiconductors exhibit two types of behavior that defines the interactions between the material and the incoming gas under study. When the formation of electron holes is the dominant carrier mechanism, the MPc exhibits a “p”-type behavior. In this case, the MPc acts as a positive charge carrier with an electron deficiency. Conversely, MPcs may exhibit an “n”-type behavior when electrons are the responsible carriers. In this scenario, the MPc acts as a negative charge carrier with an excess of electrons [8,9]. Non-substituted metal phthalocyanine (MPc) compounds typically exhibit p-type behavior, while incorporating electron-withdrawing substituents to the peripheral (α) or non-peripheral (β) positions of the MPc results in “n”-type behavior. For instance, a study conducted by Klyamer et al. investigated the effects of adding the electron-withdrawing fluorine to a CoPc structure. This modification resulted in a higher response toward ammonia (NH_3_) compared with the non-substituted analog [10]. Another study by Renjie et al. examined the impact of different groups added to the macrocyclic structure using electrochemical methods. Alkoxy groups at either position (α or β) exhibited electron-donating behavior, whereas groups such as phenyloxy showed electron-withdrawing behavior [9]. Understanding the effect when adding substituents to the macrocycle and/or the effect when varying the metal center of the structure provides insight into the mechanistic aspects of the MPc and the incoming target gas being evaluated. Previous work published by our research group also showed that the presence of fluorine substituents on the peripheral/non-peripheral positions of FePc (F16FePc)- and CoPc (F16CoPc)-based nanowires (NWs) provides selectivity toward the NH3-reducing ammonia gas when compared with their non-substituted counterparts that did not show a response [7]. 

In addition to the chemical composition, the nanofabrication of the material is also a crucial factor to consider. It directly affects the sensor’s response, interactions with the incoming gas and saturation. Optimization of the gas detection performance of the nanomaterial is also determined by its morphological structure. [11] There were several studies that showed that the greater the specific surface area and porosity of the surface, the more active sites there were. As a result, enhanced interactions between the material and the incoming gas are facilitated due to rapid transfer of electrons [12,13]. Thin films based on oxides have been developed for gas-sensing applications, as evidenced by various studies [14,15,16,17]. However, limitations of the thin-film metal-oxide-based gas sensors include requiring additional doping to enhance the response time and dependence of high temperatures for optimal functioning [18]. 

Besides the extensive reports on gas sensing using MPc thin films, nanowires (NWs) are an alternate nanoscale structure that offers a higher surface-to-volume ratio and improved crystalline order, with excellent performance and stability [7,13]. NWs growth can be achieved via physical vapor deposition (PVD). The adjustment of parameters such as the temperature and deposition period can have a direct impact on the size and morphology of the NWs. The effect on the temperature of the deposition/location of the substrate was evaluated by Tong et al. using organic vapor-phase deposition (OVPD) for the growth of CoPc, CuPc, FePc, NiPc and ZnPc nanoribbons. During this study, the authors observed twisted and straight nanostructures of the MPcs. Multiple phases were also obtained, where α phases (twisted) were achieved under lower temperatures (<119 °C) for crystalline growth, while β phases obtained after an additional annealing process showed mostly a straight arrangement (>190 °C) [19]. Other materials, such as telluride-oxide-based NWs were reported by Shen et al. for the detection of NO_2_ with at a gas concentration of 10 ppm [20]. During this study, the authors evaluated the material’s performance and observed a faster response (160%/10 s) in contrast with other materials, such as WO_3_ nanorods (202%/96 s) [21]. As previously mentioned, since oxides tend to require heating for enhanced performance, the optimal temperature for the TeO_2_ NWs was 50 °C [19].

Palladium, which is a versatile metal with exceptional properties, has garnered significant attention for its potential to revolutionize the field of highly sensitive gas sensors. Extensively researched and shown to excel in various applications, including electrochemical CO_2_ reductions and hydrogen gas sensors, palladium stands out as a promising candidate for advancing sensor technology [22]. Furthermore, incorporating Pd on the surface of diverse materials results in the optimization of performance [23]. For example, work by Liu et al. showed the benefit of incorporating palladium on vanadium dioxide (VO_2_) for NO_2_ gas detection. The authors observed that the response percentages varied between 5 to 45, approximately, during exposure concentrations between 1 ppm and 100 ppm [23]. In another study, Liangruksa et al. explored the effect of Pd in ZnO layers for hydrogen gas sensing, confirming the enhancement of the gas’s response to H_2_ [24]. However, incorporating multiple materials complicates the preparation of a gas sensor device. 

Palladium phthalocyanine (PdPc) thin films were studied as PdPc/Pd bilayer structures for hydrogen gas sensing. An improvement in the hydrogen-sensing response was observed. However, when other gases were evaluated, such as NH_3_, CO_2_, chlorinated alkane and NO_2_, no response was observed, suggesting a high selectivity toward hydrogen [25]. An additional study showed that palladium phthalocyanines (PdPc) thin-film response toward H_2_ exhibit a similar Pd behavior toward hydrogen when being coordinated in the MPc center [26]. As previously mentioned, NW structures show exceptional properties with great potential in superior gas-sensing applications. PdPc NWs are a great alternative for a maximal sensing performance device that can be combined with optimal nanomaterial fabrication. Herein, we present the development of a highly stable, facile-preparation, cost-effective and highly sensitive unsubstituted PdPc nanowire-based sensor with a response toward NO_2_ at low sub-parts-per-million (ppm) concentrations and works under normal temperature conditions. The PdPc was synthesized by a one-step solid-state cyclotetramerization reaction [27]. The NWs were deposited on interdigitated gold electrodes (IDEs) by physical vapor deposition (PVD) [7,28].

## 2. Materials and Methods

The synthesis of palladium phthalocyanine (PdPc) followed the procedure reported by Parkhomenko et al. [26]. Commercially available 1,2-dicyanobenzene was mixed with palladium (II) chloride (4:1). The solid mixture was heated at 250 °C for six hours [26]. A blue powder was obtained and purified by Soxhlet extraction using acetonitrile as the solvent. The purified product was dried at 100 °C overnight. After purification, the powder was characterized by UV–Vis spectroscopy, X-ray powder diffraction, Raman spectroscopy and energy-dispersive X-ray spectroscopy (EDS). 

**Scheme 1:**
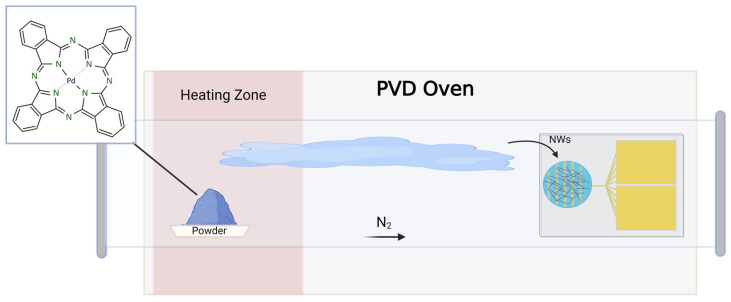
Physical vapor deposition oven for the PdPc nanowires’ growth on the interdigitated gold electrode.

PdPc nanowire formation and preparation of the sensor device was performed using physical vapor deposition (PVD) by following the procedure previously reported by our research group (Scheme 1) [7]. During the deposition process, the purified powder of the palladium phthalocyanine was used as the precursor. The solid was vaporized, followed by deposition of the material on a desired surface [7,28]. To grow the PdPc nanowires on the interdigitated gold electrode, ~15 mg of the purified PdPc powder was weighed on a ceramic holder and put into a quartz tube (1.2 m long/5 cm diameter) reactor placed inside a dual oven (MTI Corporation; CA, USA, Model OTF-1200X-II). Using a mechanical pump (Trivac D 16 B), a pressure of 5 Torr was achieved and kept constant while being monitored with an Inficon PSG55x Vacuum Gauge. 

A constant flow of nitrogen (UHP 99.999%) was passed through the tube during the deposition experiment. The temperature of the reactor was raised to 120 °C for 20 min, followed by an increment of 4.7 °C per minute to reach a temperature of 440 °C, which was kept constant for 100 min. Finally, the reactor was cooled down until reaching the laboratory temperature (~21 ± 1 °C). PdPc nanowires were also deposited on aluminum substrates for structural characterizations. 

**Scheme 2:**
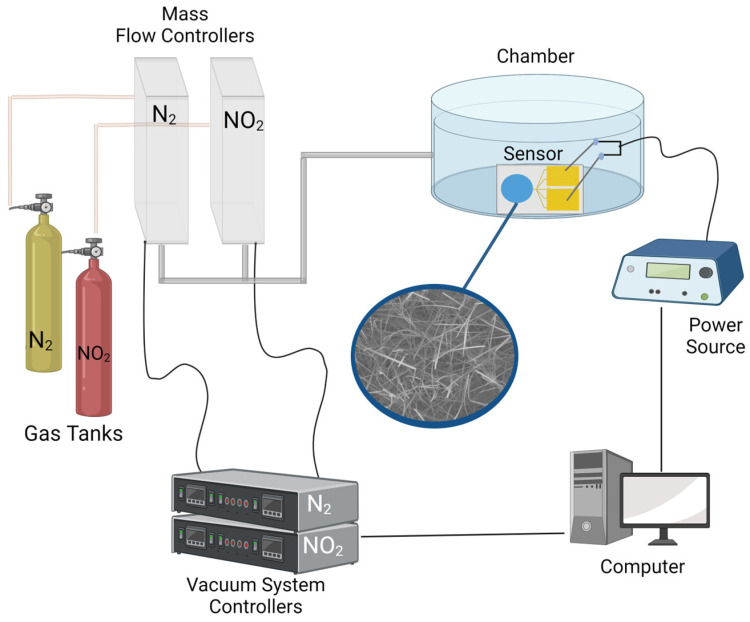
Gas-sensing experimental set up for the PdPc nanowires on the interdigitated gold electrode.

Gas-sensing experiments were performed by placing the PdPc nanowires sensor inside a gas chamber system (MMR Technologies LTMP). The device was electrically connected using two adjustable tungsten tips. A power source (Keithley 6487 Picoammeter/Voltage Source) was used to apply a constant voltage to the sensor while simultaneously measuring the current of the material. The required gas flow for each experiment was controlled by an MKS GE50A Mass Flow Controller and monitored by an MKS 946 Vacuum System Controller (Scheme 2). A constant flow of dry N_2_ gas was used to create an inert atmosphere inside of the gas-sensing chamber, similar to a previously published procedure by Yan et al. [29]. The specific target gas volume concentration was calculated using the ratios of the diluting gas (N_2_) mixed with the target gas (100 ppm NO_2_/N_2_ gas tank), producing a total gas flow of 500 sccm, and no additional humidity was added. The water presence was determined from the nitrogen tank and was <2 ppm.

The temperature inside of the chamber during the sensing experiments was 21 ± 1 °C. The sensing response of the device was analyzed by measuring the electrical current as a function of time and evaluating the change between before exposure to nitrogen dioxide (under N_2_) and afterward (N_2_/NO_2_). The normalized response parameter (*S*) is defined by Equation (1):

(1) $$S=\frac{\left|I-I_{0}\right|}{I_{0}}$$

where *I* is the current and *I_0_* the initial current before exposure to NO_2_ (baseline). 

## 3. Results and Discussion

### 3.1. UV–Vis Spectroscopy

Metal phthalocyanines can be characterized by using spectroscopic absorption studies, such as UV–Vis spectroscopy. Absorptions are attributed to electrons undergoing π → π* transitions from the highest occupied molecular orbital (HOMO) to the lowest unoccupied molecular orbital (LUMO) of the metal phthalocyanine. MPcs show very distinctive bands when coordination on the center of the structure is successful [8]. The Q-bands are the result of transitions occurring from the a_1u_ → e_g_ orbital and are usually visible between 650 and 670 nm. Two bands rise because of the lower symmetry when a metal center is coordinated in the structure. Transitions from a_2u_ → e_g_ orbitals produce the B-band that appears between 320 and 330 nm [8,30]. Characterization of palladium phthalocyanine using UV–Vis spectroscopy (Figure S1) was conducted by diluting the PdPc powder on dimethylformamide (DMF). The expected Q-bands were observed at 607 nm and 659 nm and the B-band appeared at 336 nm, confirming the coordination of the metal in the phthalocyanine.

### 3.2. X-ray Diffraction

X-ray diffraction (XRD) characterizations of the PdPc powder (precursor) and PdPc NWs are shown in (Figure S2). Previous reports of PdPc structures showed evidence of the possible formation of different phases (α, β, γ) [31]. The 2θ values for the PdPc powder showed Bragg peaks at 6.62°, 7.26°, 9.44°, 15.61°, 21.45°, 23.83°, 24.96° and 26.31°. The PdPc nanowires pattern 2θ values were 6.68°, 7.29°, 9.58°, 10.23°, 15.72°, 21.46°, 24.01°, 25.01°, 26.39°, 28.17° and 30.07°. The 2θ peaks of the PdPc powder and nanowires (6.62° and 7.26°, and 6.68° and 7.29°, respectively) suggested the formation of the α phase of the PdPc. During the deposition process, the material is heated to elevated temperatures (>400 °C), and a change in phase may occur [32]. Since there was no apparent difference on the XRD patterns intensity and position, no change in phase was observed in the pattern after the PdPc NWs deposition. Additionally, peaks were observed with higher intensity on the PdPc NWs, which provides evidence of higher crystallinity on the deposited material. 

### 3.3. Raman Spectroscopy

Raman spectroscopy is of extreme value for studying the molecular vibrations of the MPc molecules and identifying the unique bands associated for each compound. MPc Raman spectroscopy shows the vibration of the diverse atomic bonds within the macrocyclic structure [33]. In addition, the presence or absence of peripheral/non-peripheral substituents influences the bands observed in the spectrum. For instance, Klaymer et al. reported the effect of fluorine substituents on the vibration spectral of different MPcs. For further investigation, multiple MPcs, such as PbPc, ZnPc and PdPc, were compared with their fluorinated metal phthalocyanine analogue [33]. The Raman shifts (cm^−1^) of the PdPc powder and nanowires can be observed in Figure S3. According to previous reports, the distinctive shift (cm^−1^) of the band for the metal center bonding is observed between 1350 and 1550. In our case, the expected Pd band shift was observed at 1518 cm^−1^ [34].

### 3.4. Morphology and Elemental Analysis

**Figure 1:**
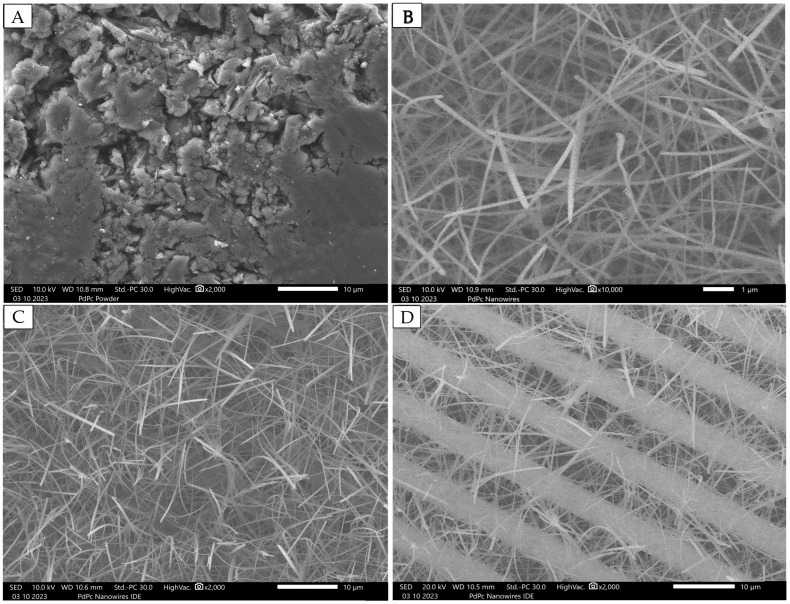
SEM images of (**A**) purified PdPc powder, (**B**) higher magnification of as-grown nanowires, (**C**) lower magnification of as-grown nanowires and (**D**) a section of the as-prepared sensor. The interdigitated electrodes are observed below the nanowires in (**B**,**C**), the substrate is uniform aluminum.

Images (Figure 1) of the scanning electron microscopy (SEM) for the PdPc materials were retrieved from different surfaces for the morphological analysis. Figure 1A shows the SEM image of the PdPc precursor powder. The PdPc nanowire growth was confirmed on aluminum substrates (Figure 1B) and deposition on the interdigitated gold electrode (Figure 1C,D). The thickness of the nanowires varied between around 0.04 μm and 0.36 μm (Figure S4). The lengths of the PdPc NWs were between 7.3 μm and 17.7 μm (Figure S5). The growth of a nanoflower was also observed on an IDE corner (Figure S6). These nanostructures have been reported for other materials and are believed to form by aggregation of the precursor material during the process of deposition [35,36,37,38]. However, it was outside of the electrically connected deposition zone of the device and did not contribute to the current response of the sensor. 

Energy-dispersive X-ray spectroscopy (EDS) provides information on the elemental composition on the material under study. The EDS of the PdPc (C_32_H_16_N_8_Pd) NWs on the interdigitated gold electrode (Figure S7) showed mass% values of C: 51.78 ± 0.05, N: 14.64 ± 0.13, O: 7.73 ± 0.06, Si: 5.67 ± 0.02, Pd: 3.54 ± 0.02 and Au: 16.64 ± 0.05. The presences of Si and Au were attributed to the IDE platform. Similarly, the EDS of the PdPc NWs on the aluminum substrate (Figure S8) showed mass% values of C: 43.10 ± 0.11, N: 5.40 ± 0.09, O: 0.56 ± 0.02, Al: 49.67 ± 0.06 and Pd: 1.27 ± 0.02. Finally, the EDS of PdPc NWs powder (Figure S9) showed mass% values of C: 51.08 ± 0.04, N: 32.20 ± 0.18 and Pd: 16.72 ± 0.05. All sample mass% values confirmed the presence of PdPc. 

### 3.5. Gas-Sensing Response

Gas-sensing experiments were conducted with an initial stabilization process under nitrogen (N_2_) with a constant flow of 500 sccm prior to gas exposure. This process allowed the excess of moisture and oxygen present in the gas-sensing chamber to exit, avoiding additional interferences that would influence the gas-sensing evaluation. During the stabilization process, the sensor was electrically connected with two tungsten tips while a constant voltage of 5 V was applied under inert conditions/room temperature for 100 min. For the sensing experiments, we selected 5 V as the optimal applied voltage by considering the optimal current values provided by the instrument when applying different ranges of voltages (1 V, 5 V, 7 V) to the IDE. 

**Figure 2:**
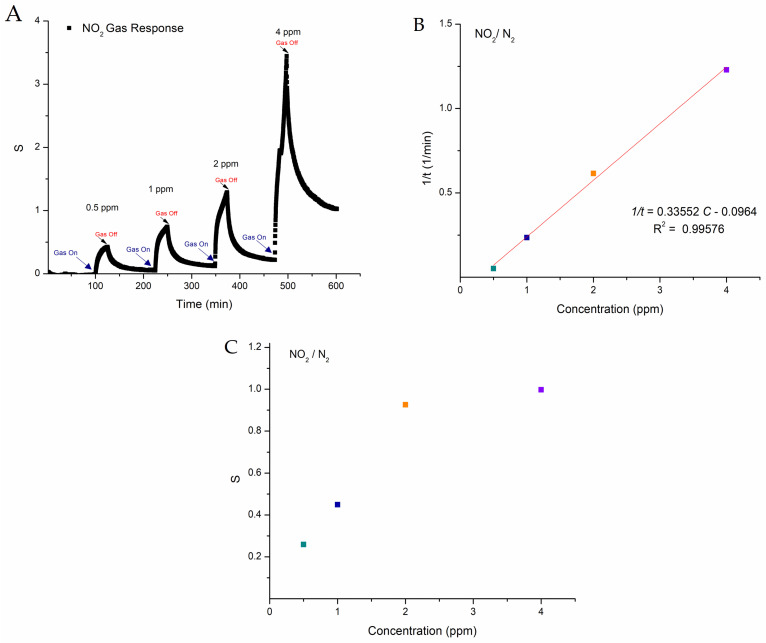
(**A**) Normalized gas-sensing response toward NO_2_ at concentrations of 0.5, 1, 2 and 4 ppm. (**B**) Inverse of the fast response time constant (1/t) as a function of concentration (ppm). (**C**) Normalized response as a function of concentration.

For the gas-sensing analysis, multiple experimental designs were performed to evaluate the factors related to the behavior of the sensor during exposure to increasing concentrations, consistency of the response after being exposed to the same concentration (0.50 ppm) for multiple cycles and reproducibility by evaluating response values to each concentration in separated experiments (Table S1). The normalized gas-sensing response (*S*) of the PdPc NWs toward NO_2_ is shown in Figure 2A. After a 100 min stabilization period, the sensor was exposed to a constant flow of NO_2_ using the appropriate gas mixture (N_2_/NO_2_) ratios to produce a concentration of 0.5 ppm inside the chamber. Testing of the target NO_2_ at 0.5 ppm was conducted for a period of 25 min, followed by a recovery process under N_2_ for 100 min. Four cycles of NO_2_ exposure were undertaken by starting with 0.5 ppm, 1 ppm, 2 ppm and 4 ppm. The change in the S value confirmed the evident correlation of the sensor’s response when under the presence of the target gas. The observed normalized response percentages for each NO_2_ gas exposure were 0.5 ppm—45%, 1 ppm—77.5%, 2 ppm—132.2% and 4 ppm—348.5%. Previous work reported by our group evidenced a similar response percentage of “n”-type substituted F_16_FePc (0.5 ppm—25%) and F_16_CoPc (0.5 ppm—8%) NWs toward the reducing NH_3_ gas at 0.5 ppm, showing superior performance when compared with thin films of other fluorinated MPcs [7,25]. Conversely, unsubstituted MPc sensors based on thin films (CuPc) showed a normalized response value of <10% toward NO_2_ at an exposure concentration of 1 ppm [38]. In addition, another oxide-based TeO_2_ NWs sensor showed a response toward NO_2_ of 160% (10 ppm) at a high working temperature (50 °C) [20]. Our response results show that the PdPc NWs possessed great sensitivity with high response percentage values (45–348.5%) toward NO_2_ at low concentrations without requiring additional heating for enhanced performance and/or sensing recovery. 

In terms of the semiconducting behavior, the PdPc NWs were expected to act as a “p”-type semiconductor in agreement with other non-substituted thin films of MPcs (CuPc, CoPc, PdPc) [26,39,40]. When “p”-type semiconductors are exposed to oxidant gases (NO_2_, NO^−^), a change in current is indicative of a response toward the incoming oxidant gas. The gas-sensing mechanism involves the PdPc NWs acting as the electron acceptor while NO_2_ acts as the electron donor [8,41]. During our different experiment designs, the PdPc NWs sensor consistently showed increasing values of current (provided by the instrument) when being exposed to the oxidant NO_2_ gas, confirming a “p”-type semiconductor behavior, as expected. 

In our previous work on F_16_FePc nanowire-based sensors exposed to ammonia [7], we identified two main contributions to the time response to the reactive gas: a fast response related to the rapid adsorption of the gas molecules to the directly exposed surface of the material and a slower one controlled by the diffusion of the incoming target gas molecules through reduced nanowire interfaces, surface porosity and extended defects. Contributions for the fast response were described using the Langmuir approximation, which assumes that the adsorption kinetics occur on the surface of the material when the incoming gas interacts with the active sites [7]. 

To confirm similar time response characteristics in the presented PdPc NWs sensors, an approximated fast response time constant was obtained (saturation~0.4) from the fast response saturation S value in the 0.50 ppm case. An overall linear dependence on the reactive gas concentration of 1/t, where “t” is the fast response time constant, was confirmed, as shown in Figure 2B, in which the concentration dependence was compared with a linear fitting. The R^2^ value of the linear fit was 0.99576. Such behavior is consistent with the target gas diffusion mechanisms proposed for F_16_FePc and F_16_CoPc nanowires-based sensors within the Langmuir approximation [7]. Within the same analysis, Figure 2C shows the sensor response (*S*) as a function of the target gas concentration measured at an exposure time of 9 min. This time was selected from the higher gas concentration (4 ppm) measured data and corresponded to the change in slope for the time in which the slow response mechanism appeared to start dominating the time dependence of the sensor response with a 4 ppm gas concentration. The S dependence on concentration at this selected time confirmed the saturation of the fast response time process and the proposed response model, as defined in Ref [7].

**Figure 3:**
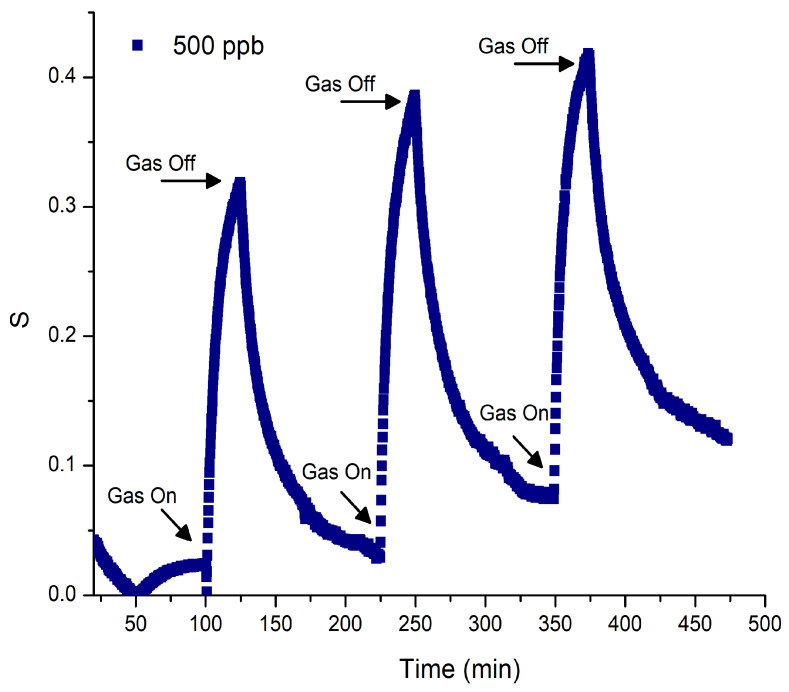
Normalized (*S*) gas-sensing response of PdPc NWs toward NO_2_ at 0.5 ppm for three cycles.

The sensor successfully detected concentrations of NO_2_ in the sub-parts-per-million range at 0.50 ppm. To evaluate the reproducibility of the results, a sensing experiment involving multiple cycles of a 0.50 ppm concentration of NO_2_ was performed. The normalized gas-sensing response (*S*) as a function of time is shown in Figure 3. Similar to the previously stated experiments, stabilization was obtained during a period of 100 min under N_2_, followed by sensor tests for NO_2_ at 0.50 ppm. The NO_2_ exposure time for each cycle was ~25 min, followed by a recovery process of 100 min between each cycle and afterward. The normalized (*S*) response percentage values for each cycle were 32.1, 38.8 and 42. Our results confirm that the sensor will consistently show high and reproducible responses toward NO_2_ at concentrations of 0.50 ppm, providing evidence that re-utilization of the device is possible. Finally, for further reproducibility analysis, multiple PdPc NWs sensor experiments were conducted for the different concentrations and are summarized in Table S1.

## 4. Conclusions

In this work, we successfully developed a palladium phthalocyanine nanowire sensor with detection in the sub-parts-per-million range, with stable detection at 0.50 ppm of NO_2_. The interdigitated gold electrode (IDE) sensors were prepared using a physical vapor deposition technique involving previously synthetized and purified PdPc powder as the precursor. Characterization to confirm the coordination of palladium in the center of the structure was achieved using UV–Vis spectroscopy analysis. Morphological studies confirmed the presence of the PdPc NWs on the IDE, uniformity of the nanostructures and growth of a PdPc nanoflower. The EDS also provided confirmation of the atomic components of the PdPc nanomaterial. The XRD patterns showed the presence of the α phase on both PdPc powder and nanowires with no indication of change in the phase after deposition. The Raman spectroscopy showed the expected band for the coordination of palladium in both the powder and NWs. 

Gas-sensing experiments were conducted with exposure concentrations of 0.5, 1, 2 and 4 ppm of NO_2_. Clear responses at various NO_2_ low concentrations were observed. Evidence of a fast adsorption process related to the directly exposed surface areas and apparent saturation on the surface was also evaluated before observing a change in the mechanism. Finally, the reproducibility of the sensing results at 0.50 ppm concentrations of NO_2_ was successfully achieved, showing similar normalized response (*S*) percentages after multiple exposures. 

## Data Availability

Data are contained within the article and the Supplementary Materials.

## Supplementary Materials

The following supporting information can be downloaded from https://www.mdpi.com/article/10.3390/s24061819/s1. Figure S1: UV–Vis spectroscopy of palladium phthalocyanine powder in DMF; Figure S2: X-ray diffraction patterns of palladium phthalocyanine nanowires and powder; Figure S3: Raman shifts (cm^−1^) of PdPc nanowires and powder; Figure S4: Thickness measurements of PdPc nanowires; Figure S5: Length measurements of PdPc nanowires; Figure S6: Scanning electron microscopy of PdPc nanoflower; Figure S7: Energy-dispersive spectroscopy of PdPc nanowires on IDE; Figure S8: Energy-dispersive spectroscopy of PdPc nanowires on aluminum substrate; Figure S9: Energy-dispersive spectroscopy of PdPc powder; Table S1: Gas-sensing experiments.
