# Supplementary material for: Palladium Phthalocyanine Nanowire-Based Highly Sensitive Sensors for NO2(g) Detection
Source: Sensors (Basel). 2024 Mar 12;24(6):1819. doi: 10.3390/s24061819 (PMC10974292; doi:10.3390/s24061819)
Supplement: Supplementary file 1 [file sensors-24-01819-s001.zip › sensors-2857367-supplementary.pdf]

Supplementary Materials

Palladium Phthalocyanine Nanowire-Based Highly Sensitive Sensors for $NO_{2(g)}$ Detection

Crystal Otero Vélez [1,2], Soraya Y. Flores [1], Luis F. Fonseca [1] and Dalice M. Piñero Cruz [1,2,*]

[1] Faculty of Natural Sciences, University of Puerto Rico, Río Piedras Campus, San Juan 00931, Puerto Rico
[2] Molecular Science Research Center, San Juan 00926-2614, Puerto Rico
* Correspondence: dalice.pinero@upr.edu

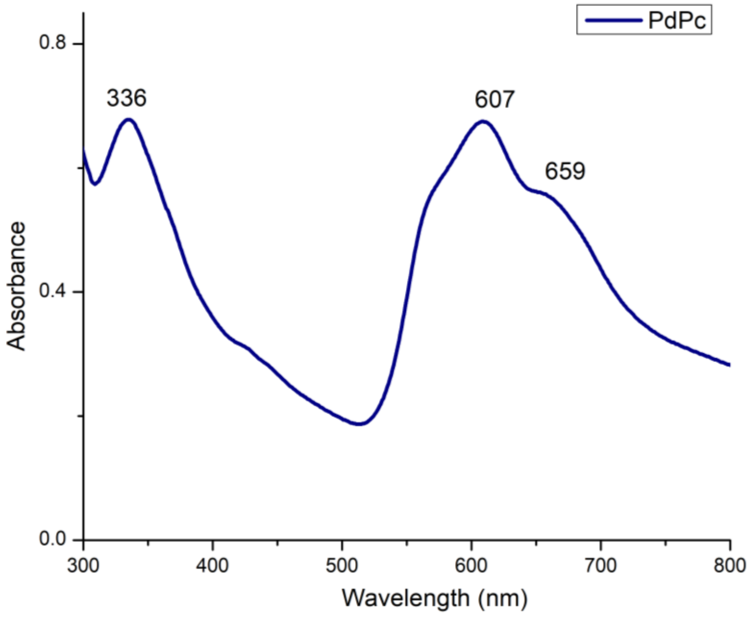

Figure S1 *Uv-vis spectroscopy of palladium phthalocyanine powder in dimethylformamide (DMF)*

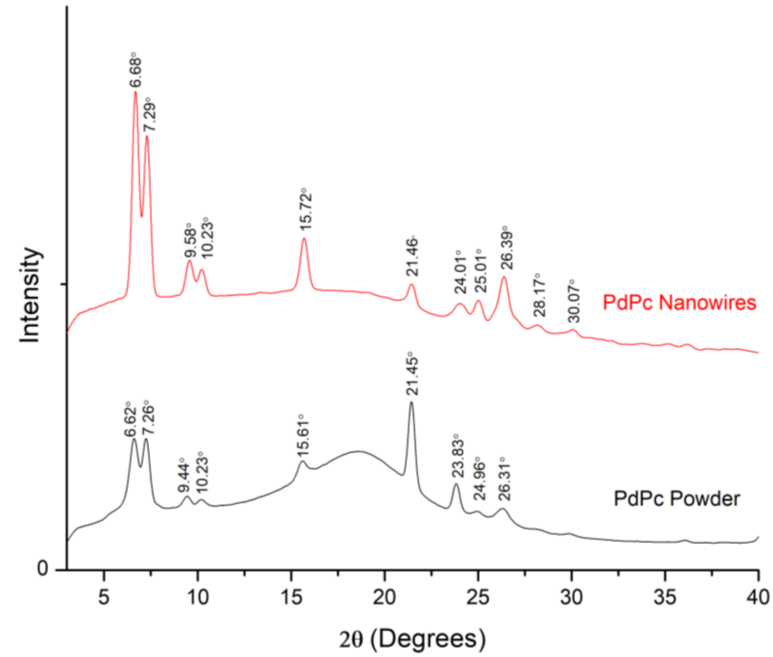

Figure S2 *X-Ray Diffraction (XRD) patterns of palladium phthalocyanine nanowires (top) and powder (bottom)*

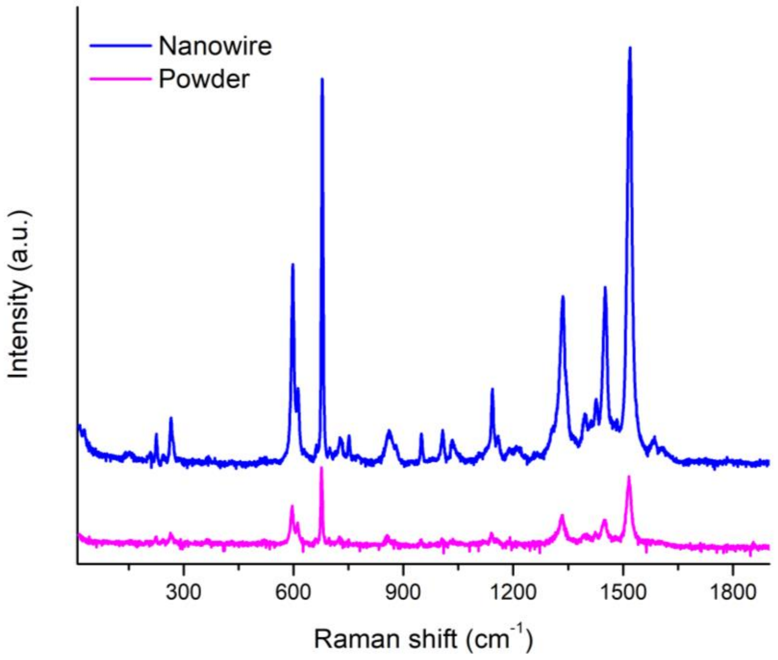

Figure S3 *Raman Shifts (cm^{-1}) of PdPc Nanowires (blue), powder (pink)*

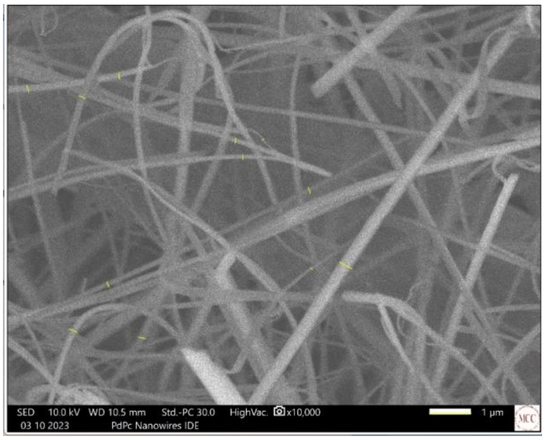

	Length (µm)
1	0.15
2	0.17
3	0.18
4	0.36
5	0.07
6	0.14
7	0.12
8	0.11
9	0.04
10	0.13
11	0.19
12	0.18
13	1

Scale Measurement

Figure S4 *Thickness measurements of PdPc NWs.*

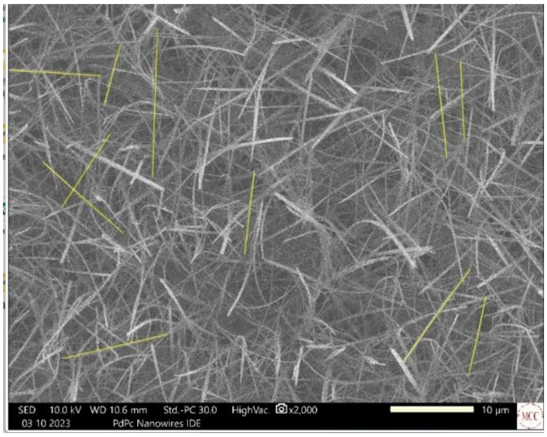

	Length (µm)
1	9.1
2	12.3
3	10.1
4	7.3
5	10.8
6	17.7
7	10.3
8	12.8
9	12.7
10	13.6
11	9.4
12	10

Scale Measurement

Figure S5 *Length measurements of PdPc NWs.*

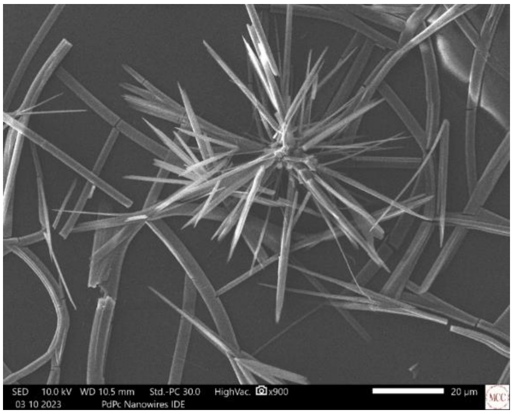

Figure S6 *Scanning electron microscopy (SEM) of palladium phthalocyanine nanoflower*

PdPc Nanowires IDE

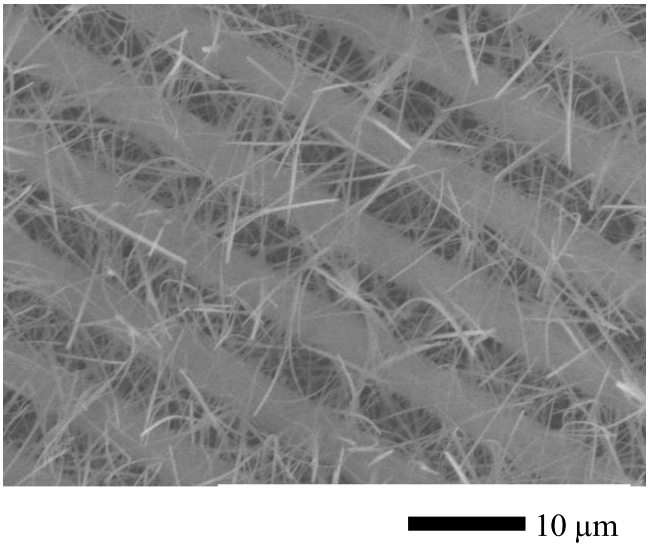

Element	Line	Mass%	Atom%
C	K	51.78±0.05	70.00±0.07
N	K	14.64±0.13	16.97±0.15
O	K	7.73±0.06	7.84±0.06
Si	K	5.67±0.02	3.28±0.01
Pd	L	3.54±0.02	0.54±0.00
Au	M	16.64±0.05	1.37±0.00
Total		100.00	100.00
PdPc Nanowires IDE		Fitting ratio 0.0754	

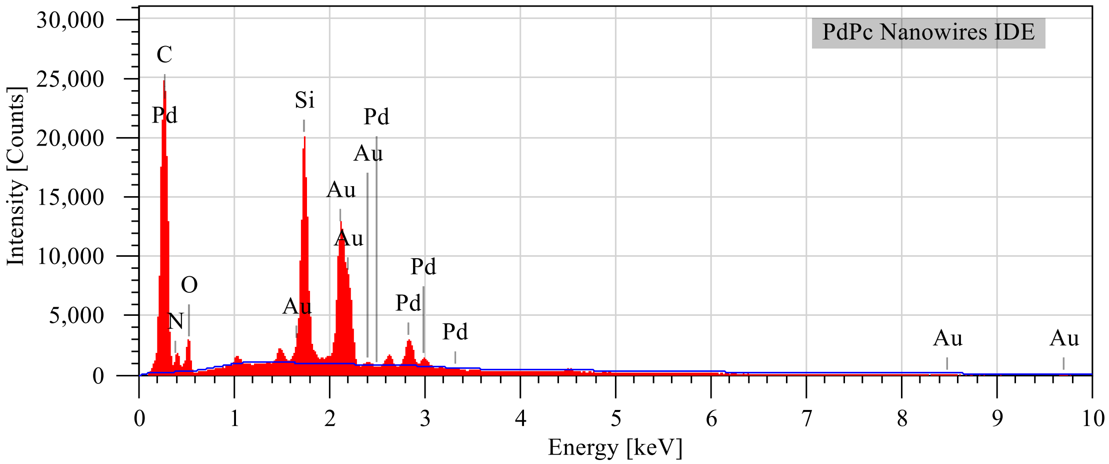

Figure S7 *Energy dispersive spectroscopy (EDS) analysis of PdPc nanowires on interdigitated gold electrode (IDE)*

PdPc Nanowires

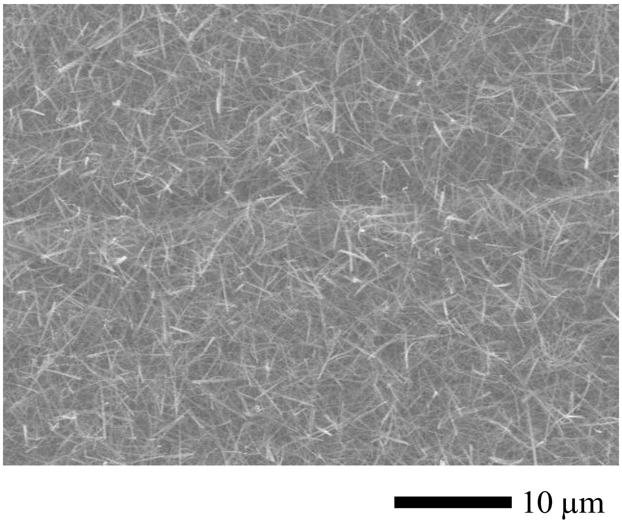

Element	Line	Mass%	Atom%
C	K	43.10±0.11	61.21±0.15
N	K	5.40±0.09	6.58±0.11
O	K	0.56±0.02	0.60±0.02
Al	K	49.67±0.06	31.41±0.04
Pd	L	1.27±0.02	0.20±0.00
Total		100.00	100.00
PdPc Nanowires			Fitting ratio 0.0063

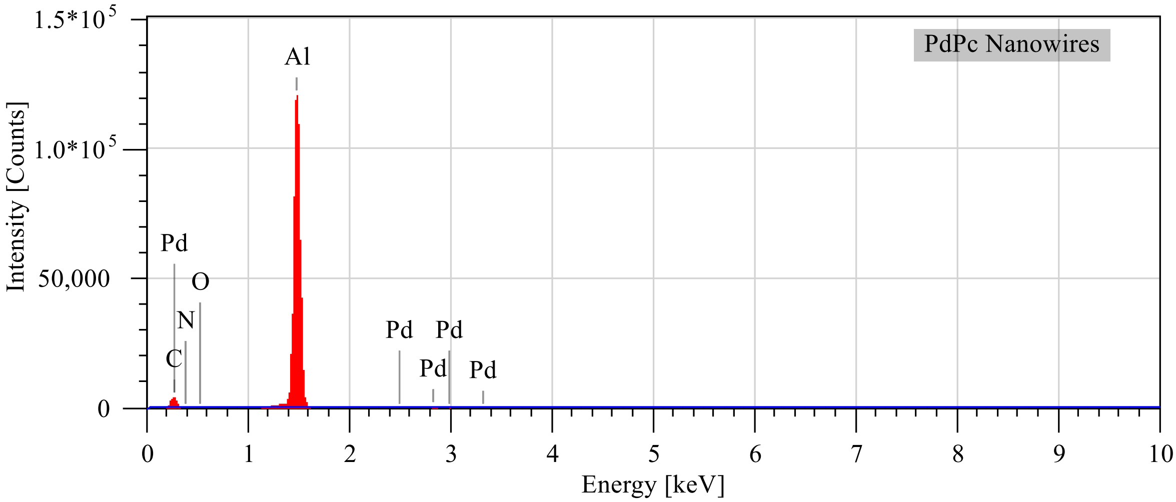

Figure S8 *Energy dispersive spectroscopy (EDS) analysis of PdPc nanowires on aluminum substrate.*

PdPc Powder

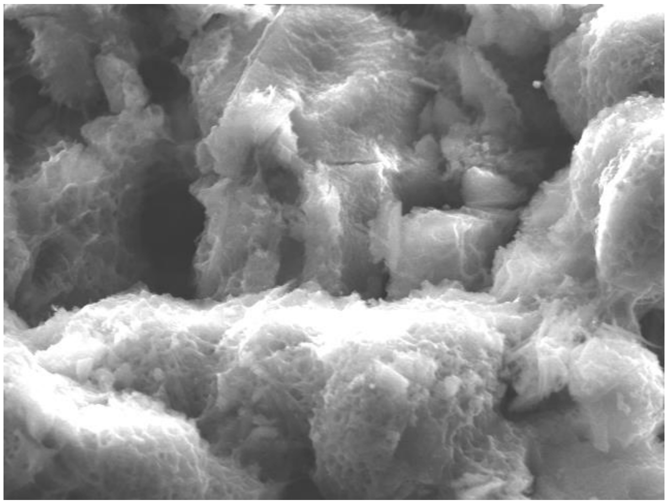

Element	Line	Mass%	Atom%
C	K	51.08±0.04	63.39±0.05
N	K	32.20±0.18	34.27±0.19
Pd	L	16.72±0.05	2.34±0.01
Total		100.00	100.00
PdPc Powder		Fitting ratio 0.1914	

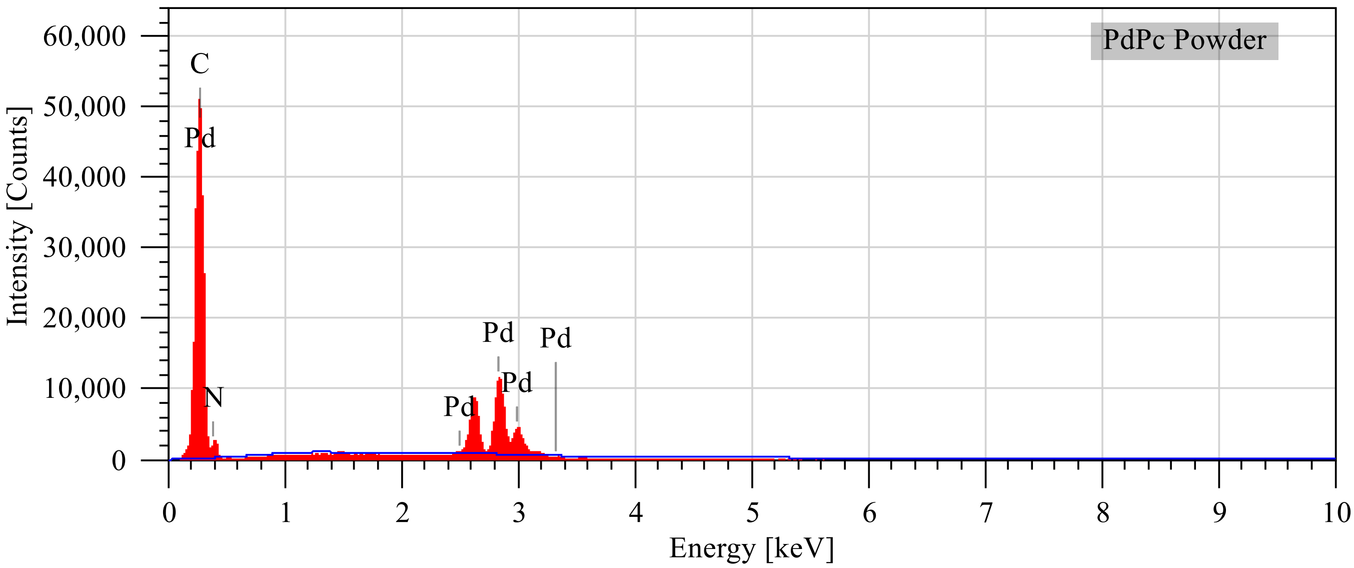

Figure S9 *Energy dispersive spectroscopy (EDS) analysis of PdPc powder.*

Table S1. Gas Sensing Experiments

Material	# of Repetitions	Tested Gas	Gas Concentration
PdPc Nanowires	8	NO_2	0.5 ppm
PdPc Nanowires	3	NO_2	1 ppm
PdPc Nanowires	4	NO_2	4 ppm
PdPc Nanowires	2	NO_2	10 ppm
